# Transcriptomics of cryophilic *Saccharomyces kudriavzevii* reveals the key role of gene translation efficiency in cold stress adaptations

**DOI:** 10.1186/1471-2164-15-432

**Published:** 2014-06-04

**Authors:** Jordi Tronchoni, Victor Medina, Jose Manuel Guillamón, Amparo Querol, Roberto Pérez-Torrado

**Affiliations:** Instituto de Agroquímica y Tecnología de los Alimentos, IATA-CSIC, P.O. Box 73E-46100, Burjassot, Spain; Departamento de Biotecnología, Instituto de Agroquímica y Tecnología de los Alimentos (CSIC), Burjassot, P.O. Box 73E-46100, Valencia, Spain

**Keywords:** *Saccharomyces cerevisiae*, *S. kudriavzevii*, Transcriptomics, Cold stress, Translation

## Abstract

**Background:**

Comparative transcriptomics and functional studies of different *Saccharomyces* species have opened up the possibility of studying and understanding new yeast abilities. This is the case of yeast adaptation to stress, in particular the cold stress response, which is especially relevant for the food industry. Since the species *Saccharomyces kudriavzevii* is adapted to grow at low temperatures, it has been suggested that it contains physiological adaptations that allow it to rapidly and efficiently acclimatise after cold shock.

**Results:**

In this work, we aimed to provide new insights into the molecular basis determining this better cold adaptation of *S. kudriavzevii* strains. To this end, we have compared *S. cerevisiae* and *S. kudriavzevii* transcriptome after yeast adapted to cold shock. The results showed that both yeast mainly activated the genes related to translation machinery by comparing 12°C with 28°C, but the *S. kudriavzevii* response was stronger, showing an increased expression of dozens of genes involved in protein synthesis. This suggested enhanced translation efficiency at low temperatures, which was confirmed when we observed increased resistance to translation inhibitor paromomycin. Finally, ^35^S-methionine incorporation assays confirmed the increased *S. kudriavzevii* translation rate after cold shock.

**Conclusions:**

This work confirms that *S. kudriavzevii* is able to grow at low temperatures, an interesting ability for different industrial applications. We propose that this adaptation is based on its enhanced ability to initiate a quick, efficient translation of crucial genes in cold adaptation among others, a mechanism that has been suggested for other microorganisms.

**Electronic supplementary material:**

The online version of this article (doi:10.1186/1471-2164-15-432) contains supplementary material, which is available to authorized users.

## Background

Nowadays, there is a trend in winemaking that consists in decreasing fermentation temperatures to improve the aromatic profile of wines. However, lowering fermentation temperatures has its disadvantages, including prolonged process duration and a greater risk of halted or sluggish fermentation [1]. These problems can be avoided by providing better-adapted yeasts to ferment at low temperature. Although the wine industry already has yeasts that are sold as cryotolerant yeasts (QA23, Lallemand Inc. or Fermol Cryophile, Fermol Reims Champagne, AEB group), most do not offer desirable fermentation performance at low temperature (10-15°C Beltran et al. [2] performed a transcriptomic analysis using the commercial *S. cerevisiae* wine-making strain QA23 during industrial fermentations at low temperature. They showed how the expression profiles during wine fermentation at 13°C contrasted significantly with those at 25°C. In particular, the genes of the cell cycle, cell growth, cell fate and maintenance categories were less expressed in the exponential growth phase at 13°C than at 25°C, whereas those genes whose expression was activated in the exponential phase of growth at 13°C were essentially those involved in the environmental stress response [3].

Previous physiological and enological works from this laboratory have already indicated the tremendous advantage of *S. kudriavzevii* fermenting at low temperature, and have shown its well-established cryotolerant character [4, 5]. Its sugar consumption rate, similar to that of *S. cerevisiae*, makes this organism a serious candidate in the wine yeast industry to compete for a place at low fermentation temperatures. The lipid composition of this species presents some features that might enable it to adapt much better at low temperature [6]. Although it has not been found in natural wine fermentations, probably due to its low ethanol tolerance, *S. cerevisiae* – *S. kudriavzevii* natural hybrids, which combine optimal characteristics of both parents, are present in cold climate wineries [6, 7]. Although we have found several genome-wide expression analysis studies that used DNA microarray technology in *S. cerevisiae*, there is no equivalent information available on other species of the genus adapting to low temperature. Therefore, the use of cryotolerant yeasts to study adaptation to low temperature can help us to better understand this stress factor and to also discriminate if these adaptation strategies are species-specific or common to all the strains of the *Saccharomyces* genus.

In this study, we conducted a comparative genome-wide gene expression analysis between a well-known wine yeast strain belonging to the species *S. cerevisiae* (T73) and the type strain from *S. kudriavzevii* IFO1802, a cryotolerant yeast, in natural must fermentations. Significant differences were found in the expression of those genes related to translation machinery. Sensitivity of translation inhibitor paromomycin reflected the enhanced translation efficiency of *S. kudriavzevii* at low temperatures. Indeed, an increased translation rate of *S. kudriavzevii* was observed after cold shock, suggesting that the efficiency of protein synthesis is an important process for the adaptation of yeast cells to grow at low temperatures.

## Results

### Effect of low temperature stress

**Figure 1:**
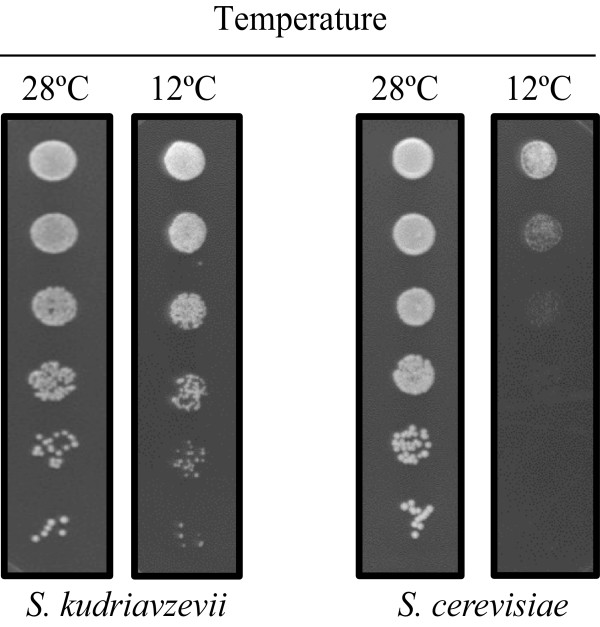
***S*** . ***kudriavzevii*** **has increased grow abilities at low temperature.** A drop test assay was performed in rich media GPY with *S. cerevisiae* strain T73 and *S. kudriavzevii* strain IFO1802 and plates were incubated at different temperatures (12 or 28°C).

Previous studies in our laboratory have clearly shown growth differences among the species belonging to the genus *Saccharomyces* in colony development on GPY plates at low temperature [8]. To confirm this behaviour in the strains selected for this study, we performed a dropping assay at 12°C with IFO1802 (*Saccharomyces kudriavzevii*) and used the yeast T73 (*Saccharomyces cerevisiae*) (Additional file 1: Table S1) as a control to verify the enhanced growth of the *S. kudriavzevii* strain at the selected cold temperature. Colonies were grown on GPY plates incubated at 12°C and 28°C to test yeast tolerance against low temperature (Figure 1). Growth at 28°C was recorded after 36 h of incubation, while growth at 12°C was recorded after 6 days of incubation. At 28°C, both strains were able to grow until the last dilution. At 12°C, IFO1802 reached the last dilution, while T73 was clearly at a disadvantage since it displayed a strong low temperature effect.

Differential growth kinetics was also observed during wine micro-fermentations in vessels with 0.45 L of Tempranillo grape must. At 12°C, the *S. kudriavzevii* strain took around 50 hours to consume 15% of sugars, almost 3 times less than T73, which demonstrates the better adaptation of IFO1802 to low temperature. In addition, *S. kudriavzevii* strain maintained a good fermentation ratio until the end (11 days). T73 finished in 21 days (Table 1). Thus, these data not only prove that the selected temperature produces a cold stress in both yeasts, although it becomes much more critical in T73, and but also validate this temperature to produce differences in the adaptation of both strains to cold temperature.

**Table 1:** **Time needed to consume 15**% **(T**_**15**_ **), 50**% **(T**_**50**_ **) and 100**% **(T**_**100**_ **) in Tempranillo must micro fermentations**

		12°C			28°C		
specie	strain	t_15_^1^	t_50_^1^	t_100_^2^	t_15_^1^	t_50_^1^	t_100_^2^
*S. cerevisiae*	T73	129.9^b^	210.7^b^	21^b^	7.9	35.8	6
*S. kudriavzevii*	IFO1802	51.8^ab^	94.5^ab^	11	20.1^a^	38.3	11^b^

^1^Hours, ^2^days. ^a^Significant differences compared to the control strain (T73) at the same temperature. ^b^Significant differences due to temperature.

### Differential gene expression in *S. cerevisiae* and *S. kudriavzevii* at low temperature

Changes in the global expression of genes during acclimation to low temperature in the wine fermentation of natural must were analysed with microarrays containing the annotated genome of the *S. cerevisiae* S288c strain. This comparative transcriptomic study of *S. cerevisiae* and *S. kudriavzevii* was carried out during wine micro-fermentations in 500 mL vessels of Tempranillo grape must at 12°C and 28°C with wine yeast strain T73 and IFO1802. The gene expression of these species was analysed at the beginning of the exponential phase by taking samples two generations after inoculation. After extracting RNA and verifying quality, the samples were retrotranscribed to cDNA, and were mixed in equal amounts and hybridised against the microarray based on the S288c genome. It should be borne in mind that an average of 86% of sequence similarity exists between *S. cerevisiae* and *S. kudriavzevii*. Thus, we used heterologous conditions in the hybridisation of the cDNA of both strains tested against the *S. cerevisiae* S288c microarrays. Genomic DNA hybridisations were done previously to assure the efficiency of the methodology; under our conditions, 95% of the total gene spots from the S288c array were hybridised by *S. kudriavzevii* DNA [9]. The use of non-restricted conditions increases the noise, but improves the hybridisation of the *S. kudriavzevii* samples. The gene expression of each strain was compared at both temperatures by performing biological triplicates. Significant genes with differential expressions were taken into account for further analysis using the SAM (Significance Analysis of Microarrays) test with an FDR below 5%. Table 2 shows the amount of the up- and down-regulated genes found in each species in relation to temperature and after comparing one species against the other. In *S. cerevisiae* strain T73, 177 genes were up-regulated, while 194 were down-regulated at 12°C, while for *S. kudriavzevii,* these numbers were 160 genes and 128, respectively. The transcriptome variation between temperatures (12-28°C) in *S. cerevisiae* was compared with the transcriptome variation between temperatures (12-28°C) in *S. kudriavzevii,* and an increased expression of 231 genes in IFO18802 and of 78 in T73 was observed (Additional file 2: Table S2).

**Table 2:** **Functional group analysis of transcriptomic data**

			GO terms			
Sample	Regulation	No. of genes	Name	No. of genes	***p***-value	Example genes
	Up	177	Cytoplasmic translation	20	2.5 · 10^−6^	TIF1:SUI1:RPL19B:RPS18B
T73 12–28°C			Localization	54	9.8 · 10^−3^	ERP2:BSD2:GLE1
	Down	194	-	-	-	OYE3:FLO1:STI1:LIP1:IMH1
	Up	160	Translation	68	9.7 · 10^−26^	TIF1:EFT1:SUI1: RPL19B:RPS8A
			Amino acid catabolic process via Ehrlich pathway	10	9.8 · 10^−4^	PDC1:PDC5:ADH3:ADH2:ADH1
IFO1802 12–28°C						
	Down	128	-	-	-	OYE3:FLO1:GRE2:LIP1:CST6:IMH1
IFO1802 12–28°C	Up	231	Translation	59	8.4 · 10^−10^	TIF1:EFT1:RPL12A:RPS8A
Vs						
T73 12–28°C	Down	78	-	-	-	ADI1:SPO7:SMA1

The GO terms analysis (GO Term Finder) was performed to observe the functions of the significant up- or down-regulated genes (Table 2). At 12°C, T73 showed the GO-terms Cytoplasmic translation and localisation to be significantly up-regulated, whereas no functional group was found among the down-regulated genes. *S. kudriavzevii* also showed significantly up-regulated translation, together with the term Amino acid catabolic process via the Ehrlich pathway. Finally, the comparison made of both species between them ended up with only one significant functional group present in the IFO1802 up-regulated genes: Translation. The presence of this GO was confirmed with the FunSpec database that also detected other translation related functional groups (rRNA export from nucleus) and protein complexes (cytoplasmic ribosomal large and small subunit) (Addtional file 4: Table S4). This result suggests that, although both species increased the expression of those genes related to protein biosynthesis, *S. kudriavzevii* has an enhanced or faster response after cold shock.

In an attempt to find the putative signal transduction pathways involved in cold adaptation, we searched the transcription factors that regulate each up-regulated gene in the Yestract database [10]. In this search, we found that the main transcription factor was Sfp1p, which regulates the transcription of ribosomal proteins and biogenesis genes, and is related to 62.3% of the activated genes in IFO102 and to 49.7% of the activated genes in the T73 strain. Furthermore, Sfp1p was the principal regulator in the genes activated when comparing both species, with 50.4% of the genes. This result confirms not only the importance of the translation machinery in response to cold shock in both species, but also the enhanced response of *S. kudriavzevii*. We also found that 21.6% of the genes induced in IFO1802 and 24.5% in the T73 strain were regulated by Msn2p. This result is also interesting since the transcriptional network of the complex Msn2p/Msn4p has also been suggested to participate in adaptation to cold stress [11, 12].

### Cold stress markers in adaptation to low temperature

Several genes that have been systematically found in different transcriptomic studies in response to low temperature stress are considered cold stress markers [11–13]. One example of a gene induced by low temperature and involved in translation initiation is *NSR1*, which encodes a nucleolar protein that binds nuclear localisation sequences and is required for pre-rRNA processing and ribosome biogenesis. Among the genes regulated by Msn2p/Msn4p, a paradigm group of genes systematically found in different transcriptomic studies in response to low temperature stress are different heat shock proteins related to oxidative stress. This is the case of *HSP12,* which encodes a chaperone involved in correct protein folding under many stress conditions, including cold shock. We observed that *NSR1* and *HSP12* were up-regulated in both yeasts after cold shock (Additional file 3: Table S4), thus confirming once more its implication in the cellular response against low temperature stress.

The comparison made between both species showed an increased expression of other cold shock markers in the IFO1802 strain if compared to T73: *OLE1*, *TIP1*, *ERG1* and *PAU4. OLE1* and *ERG1* are implicated in lipid metabolism, whereas *TIP1* and *PAU4* belong to the DAN/TIR family of putative cell-wall mannoproteins and its sequence-related seripauperin (PAU) family, respectively. This observation supports the idea of an enhanced transcriptional response in *S. kudriavzevii* as compared to *S. cerevisiae* after cold shock.

### *NSR1* expression profile during synthetic must fermentation

**Figure 2:**
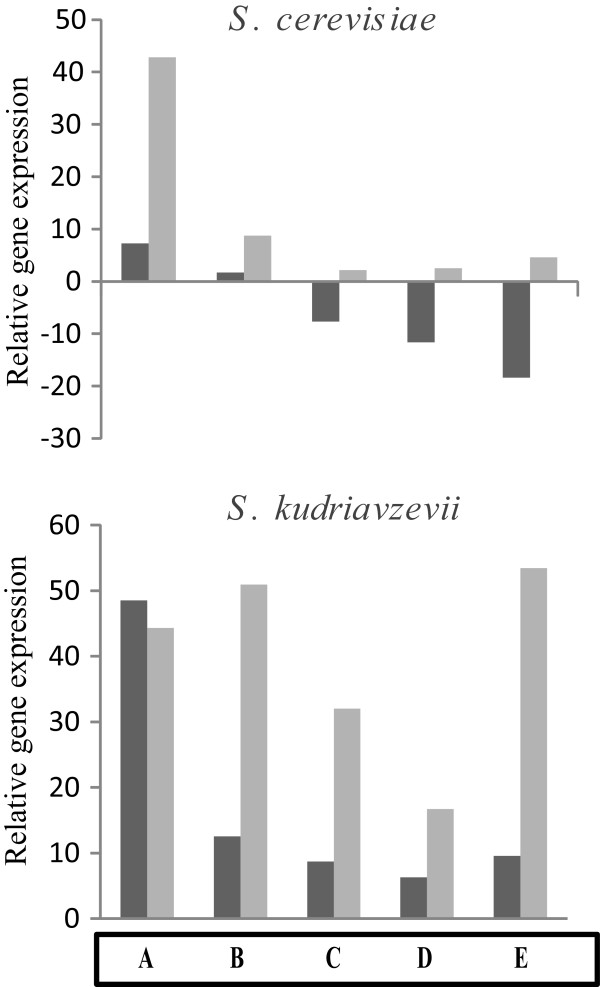
***S. kudriavzevii*** **has increased expression of the cold stress gene marker** ***NSR1*** **at low temperature.** Gene expression measured by qPCR technique after *S. cerevisiae* (T73) or *S. kudriavzevii* (IFO1802) cells were transferred to 12 (light grey) or 28°C (dark grey) synthetic grape must. Samples were taken 2 hours after the inoculation (A), when the OD_600_ reached the double of the initial OD_600_ (B), in the middle of exponential phase (C), and at the start (D) and at the end (E) of the stationary phase (sugar exhaustion). Average of biological triplicates was calculated and standard deviations were lower than 20%. Gene expression levels are shown as the changes in the concentration of the studied gene compare to the control sample and normalized with the concentration of the housekeeping *ACT1* gene. Gene expression differences between 12 a 28°C were statistically significant, except in the case of time point A for *S. kudriavzevii*.

It was our intention to test the expression profile of one of the main cold markers genes defined for cold in other time points of fermentation and to know if *NSR1* behaves similarly in both species. To do this, we did fermentations in synthetic must, a media that mimics natural must. The time points selected at both temperatures were: 2 h after inoculation, when the OD doubled the initial OD, in the middle of exponential phase, and finally at the start and at the end of the stationary phase (sugar exhaustion) (Figure 2). As previously described [14], cold marker *NSR1* in the *S. cerevisiae* strain was induced at the beginning of growth with a much higher expression level at 12°C (Figure 2). This confirms the use of this gene as a cold marker. During the entrance in the stationary phase, it was repressed at 28°C and became less activated at 12°C in *S. cerevisiae*. In *S. kudriavzevii*, there a similar induction to *S. cerevisiae* occurred, but it was not repressed as it was in *S. cerevisiae*, and maintained greater activity during the rest of the process, especially at 12°C.

### Translation efficiency at low temperature

**Figure 3:**
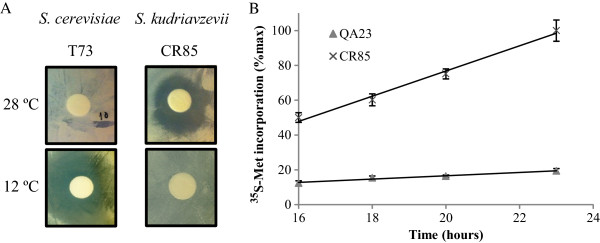
***S. kudriavzevii*** **presents increased translation efficiency at low temperatures.** In **panel A**, the inhibitory effect of the translation inhibitor paromomycin was evaluated by measuring the halo diameter generated in *S. cerevisiae* (T73) or *S. kudriavzevii* (CR85) lawns growing in GPY plates at 28 or 12°C. In **panel B**, the translation efficiency was evaluated by measuring ^35^S-Methionine incorporation 16 h after transfer *S. cerevisiae* (Q23) or *S. kudriavzevii* (CR85) cells to cold (12°C) rich media. Cpm values were normalized with OD_600_, relativized to maximum value and represented against the time. Average of biological triplicates and standard deviations are shown.

To demonstrate that *S. kudriavzevii* adaptation to low temperature is related to enhanced translation efficiency, we tested its sensitivity to paromomycin, a potent translation inhibitor [15]. We studied the paromomycin (0.4, 2 and 10 mg/ml) growth inhibition of yeast cells at either 28°C or 12°C for two *S. cerevisiae* strains (T73 and QA23) and two *S. kudriavzevii* strains (IFO1802 and CR85). A growth inhibition halo was observed under some conditions (Figure 3A). A summary of the results, presented in Table 3, indicates that *S. cerevisiae* strains T73 and QA23 were severely affected by paromomycin at 12°C whereas the *S. kudriavzevii* strains showed low (CR85) or no growth inhibition at all (IFO1802). This result confirms the better translation performance of *S. kudriavzevii* at 12°C. At 28°C, the *S. cerevisiae* strains showed no growth inhibition, whereas *S. kudriavzevii* strains presented a mild negative effect of paromomycin on cell growth. This result can be explained because 28°C is a high temperature for *S. kudriavzevii*[16] and this suboptimal situation, together with paromomycin inhibitory effects, can affect cell growth.

**Table 3:** **Sensitivity of the different yeast strains to translation inhibitor paromomycin at 28°C or 12°C**

	***S. cerevisiae***						***S. kudriavzevii***					
	T73			QA23			IFO1802			CR85		
Paromomycin (μg)	0.4	2	10	0.4	2	10	0.4	2	10	0.4	2	10
28°C	-	-	-	-	-	-	+	+	++	+	+	++
12°C	+	++	+++	+	++/+++	+++	-	+	+	-	-	-

Size of the halo produced in the lawn of yeasts was: − (0), + (0–0.5), ++ (0.5-1.0) and +++ (1.0-1.5) in cm.

Since the data obtained in this work suggested an enhanced response to cold shock in the *S. kudriavzevii* strain, especially in those genes related to translation machinery, we decided to study the translation kinetics of both species after cold shock by measuring the ^35^S-methionine incorporation rate before yeast cells started to grow (Figure 3B), which is an indication of the translation rate. Quantification of the ^35^S-methionine incorporation rate to nascent peptides after transfer yeast cells at either 28°C or 12°C culture media for two *S. cerevisiae* strains (T73 and QA23) and two *S. kudriavzevii* strains (IFO1802 and CR85) is presented in Table 4. The results show that the ^35^S-methionine incorporation rate of both *S. kudriavzevii* strains was significantly lower than any *S. cerevisiae* strain at 28°C (measured between 1–5 h after transferring cells). In contrast at 12°C (measured between 16–24 h after transferring cells), the situation was the opposite; the ^35^S-met incorporation rate of both *S. kudriavzevii* strains was significantly higher than any *S. cerevisiae* strain. These results support the notion that the cryotolerant species *S. kudriavzevii* has an enhanced translational response after cold shock, which allows cells to better adapt to the stressful condition and start to grow.

**Table 4:** **The** ^**35**^ **S-methionine incorporation rate** ^**1**^ **of the different yeast strains**

	***S. cerevisiae***		***S. kudriavzevii***	
	T73	QA23	IFO1802	CR85
28°C	54.5 ± 2.5	165.1 ± 1.9	21.2 ± 2.0*	9.6 ± 3.3*
12°C	1.1 ± 0.3	0.57 ± 0.1	2.2 ± 0.3*	4.4± 0.3*

*Significantly different (p < 0.01) to any *S. cerevisiae* strain value.

^1^Rates are expressed in CPM · UOD_600_^−1^ · h^−1^.

## Discussion

Temperature fluctuations are an inevitable aspect of microbial life in exposed natural environments, although sub-optimal temperatures are also common in industrial processes. Low temperatures (10-15°C) are used in wine fermentations to enhance production and to retain flavour volatiles. Although *S. cerevisiae* is always predominant in wine fermentations, a drop in temperature affects its competitiveness. In contrast, *S. kudriavzevii* showed the lowest optimum growth temperature, which confirms that this species is more psychrophilic than *S. cerevisiae*, and that its competitiveness increased as the fermentation temperature dropped [17–19]. Thus, despite them being absent in fermentative environments, *S. kudriavzevii* strains have a great potential for being used in wine fermentations at low temperature. Moreover, if these strains are not sufficiently stress-tolerant to cope with the stress of wine fermentation conditions, they can be used to build artificial hybrids with *S. cerevisiae* strains. These hybrids might acquire some physiological properties of both parents. It should be noted that the natural hybrids of *S. cerevisiae* × *S. kudriavzevii* have already colonised central European wine fermentations [20]. The psychrophilic nature of the *S. kudriavzevii* strain has once again been supported by the drop test obtained at low temperature in this study.

In any case, *S. kudriavzevii* is a good model to study adaptation at low temperatures.. A transcriptome comparison with *S. cerevisiae* has shed light on the response of this cryotolerant yeast species. A common answer for both species is the presence of the up-regulated genes related to translational machinery (Table 2). There are a number of publications of genome-wide analysis at low temperature [12, 13, 21–23]. As Tai et al. [13] remarked, these studies present some inconsistencies; for instance, the different expression of ribosomal protein genes. Since we selected a condition where cells were already growing at both temperatures, we did not expect any differences in ribosome biogenesis or proteins synthesis because these categories are typical from the start of exponential growth. On the contrary, our results reveal that both species have up-regulated GO-terms related to translation, although *S. kudriavzevii* shows an enhanced response. We postulate that it could be the result of changes in the stability of a functional RNA conformation in relation to a competing structure [24]. It has been described that cold sensitive phenotypes, caused by hyper-stabilisation of RNA structures, can be found elsewhere in the spliceosome, where RNA structures must form and then disrupt for splicing to progress. If this plasticity of RNA structures is reduced by RNA stabilisation due to a cold environment, the maturation of ribosomes may be in danger [24–29].

Another result that relates low temperature adaptation and translation efficiency of the *Saccharomyces* species is the differential regulation of cold shock gene marker *NSR1.* This gene, together with other nucleolar proteins with small nucleolar RNAs (snoRNAs), is required for the normal processing of rRNA precursors. These snoRNAs associate with pre-rRNA as snoRNP complexes and participate in the assembly of ribosomal subunits [30]. The plasticity of these secondary RNA structures is essential to constitute snoRNP complexes, but secondary RNA structures are highly subjected to malfunction due to low temperatures. It is possible that some of these snoRNAs are more sensitive to cold than others, especially those required by NSR1p. Studies in prokaryotes have shown the induction of a set of cold shock proteins, which include RNA helicases [31]. It is possible that *NSR1* can function as bacterial cold shock proteins (CSPs), which destabilise the RNA secondary structures that have been stabilised as an effect of low temperatures and acts as an RNA chaperone. CSPs also present greater protein resistance to unfoldment, and therefore play an important role in adaptation to low temperature [32, 33]. The interspecies differences observed in the *NSR1* gene expression profile during a fermentative process between *S. cerevisiae* and *S. kudriavzevii* are an example of how different yeasts species adapt to low temperatures.

The different susceptibility to paromomycin suggests that other mechanisms might be implicated in *S. kudriavzevii-*enhanced adaptation to low temperatures. Paromomycin, a potent inhibitor of translation, is a member of the aminoglycoside family of antibiotics. This family is thought to reduce the dissociation rate of A-site tRNA from the ribosome [34]. Paromomycin increases the error rate of the ribosome, but it has been also described as a powerful inhibitor of ‘ribosome recycling’ [35]. Ribosome recycling represents the reaction to recycle the spent ribosome for the next round of translation of new mRNA. Kurata et al. [36] have recently reported that paromomycin negatively affects all different ribosome recycling steps to produce a dramatic effect on translation efficiency. Thus, increased resistance to paromomycin can be the result of enhanced translation efficiency due to an increased number of ribosomes available to a new round of mRNA translation. Since *S. kudriavzevii* is less affected by paromomycin at low temperatures than *S. cerevisiae*, our data suggest that this species has increased translation efficiency due to higher ribosome availability after adaptation to cold shock.

It is also worth mentioning the specific activation of the genes of the amino acid catabolic process via the Ehrlich pathway. Recently our group compared the metabolomic differences between *S. cerevisiae* and *S. kudriavzevii*[37]. The main differences between the metabolic profiling of both species were observed for amino acids. In fact, nitrogen metabolism is one of the most affected cellular processes at low temperature in *S. cerevisiae*. Pizarro et al. [38] reported that the physiological and transcriptional response of laboratories and wine yeast strains to stress at low temperature was similar to growth under nitrogen-limiting conditions. Thus, the stronger activity in *S. kudriavzevii* of the genes involved in one of the main amino acid pathways might represent this metabolic bottle-neck having been better surpassed in this species. As already reported Tronchoni et al. [6], a better adapted lipid membrane composition in *S. kudriavzevii* might enable better transport of nitrogen compounds and, therefore, more activity of amino acid metabolism.

## Conclusions

Our results confirm that *S. kudriavzevii* is better adapted to grow at low temperatures and reveals an enhanced translation in *S. kudriavzevii*. Our data suggest that translation efficiency can be an important target of adaptative evolution when cells face changing environments. New studies of comparative genetics could shed light on the specific mechanism underlying enhanced translation efficiency at low temperatures.

## Methods

### Strains and media

The yeasts used in this study belong to the species *S. cerevisiae* and *S. kudriavzevii.* Additional file 1: Table S1 shows the references and origin of these yeasts. T73 (ATCC 90607) and QA23 are commercial strains, marketed by Lallemand S.A. (Canada), that have been used as a wine yeast *S. cerevisiae* model in many studies [16, 39]. IFO1802 (NBRC 1802) is the *S. kudriavzevii* type strain and CR85 was isolated in Spain [40]. Strains are available upon request. GPY medium (0.5% peptone, 2% glucose, 0.5% yeast extract) was used to propagate yeast strains.

### Natural must fermentations

Yeast strains were cultivated in Erlenmeyer flasks containing 250 ml of GPY at 25°C in an agitated incubator (Selecta, Barcelona, Spain). At the end of the exponential phase, determined by absorbance at 600 nm, 2 × 10^6^ cells ml^−1^ were inoculated in each grape must flask. Fermentations were carried out in triplicate using 450 ml of Tempranillo grape must at pH 3.5. Before fermentation, must was clarified by sedimentation for 24 h at 4°C in the presence of 60 mg l^−1^ of sulphur dioxide. After separation, chemically pure glucose and fructose were added to raise the sugar content to 250 g l^−1^. The must was then supplemented with 0.25 g l^−1^ of yeast nutrients (Lallemand, Montreal, QC, Canada). Yeast assimilable nitrogen was determined by the formol index method [41], and diammonium sulphate was added to reach a final concentration of 250 mg l^−1^. Finally, must was sterilised by adding dimethyl dicarbonate (Fluka, Buchs, St. Gallen, Switzerland) at a concentration of 1 ml l^−1^ must.

To follow wine fermentation kinetics, Tempranillo grape must was fermented at 12°C and 28°C. Fermentations were carried out in biological duplicate and monitored by sugar consumption. Glucose and fructose concentrations were determined enzymatically in duplicate using a commercial kit (AMS-SYSTEA) in an Echo-Enosys analyser (Tecnova S.A., Madrid, Spain). Fermentations were finished when the concentration of reducing sugars was lower than 2 g l^−1^.

### Synthetic must fermentations

Experiments were carried out in a complex synthetic medium (MS300) to mimic a standard natural must previously described by Bely et al. [42]. Natural musts show a variable composition among different seasons that can influence yeast growth. For this reason, a defined synthetic must was chosen in this work as the most appropriate growth medium to overcome this variation. The sugar concentration (50% glucose, 50% fructose) was adjusted in distilled water according to the previously described natural must Tempranillo and was heated at 100°C for 15 min to prevent sugar caramelization. The stocks for the other components of the medium (mineral salts, vitamins, amino acids and anaerobic factors) were previously sterilised by filtration (0.2 mm) and were then added to the basal medium at the appropriate concentration [43]. Finally, pH was adjusted by aseptically adding tartaric acid (85%, wt/vol) according to the experimental design. We chose this organic acid because it is a compound that is normally found in grapes and wines, and it is very rarely metabolised by ascomycetous yeasts. Sterile glass bottles (500 ml of volume) were filled with 450 ml of synthetic must and were independently inoculated with 50 μl of the corresponding yeast saline suspension to reach an initial concentration of inoculum of about 2 × 10^6^ cells ml^−1^ determined by absorbance at 600 nm. Bottles were incubated at 12°C and 28°C.

### RNA extraction

Cells were collected by centrifugation (4000 rpm/min, 5 min) from three independent fermentations at 12°C and 28°C at the beginning of the exponential phase by taking samples two generations after inoculation. The RNA extraction method was based on consecutive treatments with phenol-tris, phenol-chloroform (5:1) and chloroform-isoamyl alcohol (24:1), and a final precipitation with ethanol and sodium acetate [44]. RNA concentrations and purity was determined using a Nanodrop spectrophotometer ND-1000 (Nanodrop Technologies™, Wilmington, DE). RNA integrity was determined by electrophoresis in 1% agarose gel.

### Microarray hybridisation

Firstly, 2–4 μg of total RNA from each sample were linearly amplified using the Low RNA Input Fluorescent Linear Amplification kit (Agilent Technologies™, Ca, USA). 2–3 μg of amplified cRNA were used as a template for cDNA synthesis. cDNA was marked indirectly with the “SuperScript™ Indirect cDNA Labeling System” (Invitrogen™, San Diego, CA). The fluorophores used were Cy3 and Cy5 mono-reactive Dye (Amersham GE Healthcare™, Amersham, UK) and dye incorporation was monitored by a Nanodrop spectrophotometer. A mixture of 200–300 pmol of the two labelled samples was concentrated in a Concentrator Plus (Eppendorf™, Hamburg, Germany). Competitive hybridisation was performed on a Yeast 6.4 K Array, PCR-amplified ORFs of yeast S288c strain, (Microarray Centre, UHN, Toronto, Canada) in hybridisation chambers AHC (ArrayIt Corporation, CA, USA) at 42°C overnight.

The pre-hybridisation solution contained 3X SSC, 0.1% SDS and 0.1 mg/ml BSA; hybridisation solution contained 5X SSC, 0.1% SDS and 0.1 mg/ml of salmon DNA. Microarrays were washed manually with different solutions containing distinct SSC 20X and SDS 10% concentrations (Sol.1: 2X SSC-0.1% SDS; Sol.2: 0.1X SSC-0.1% SDS; Sol.3: 0.1 SSC; Sol4: 0.01X SSC). Signal intensities of Cy3 and Cy5 were acquired with an Axon GenePix 4100A scanner (Molecular Devices, CA, USA) using the GenePix Pro v.6.1 software at a resolution of 10 μm. Genomic DNA hybridisations were done previously to assure the efficiency of the methodology; under our conditions, 95% of the total gene spots from the S288c array were hybridised by *S. kudriavzevii* DNA [9]. The use of non-restricted conditions increases the noise, but improves the hybridisation of the *S. kudriavzevii* samples.

Microarray data were derived from three independent experiments for cDNA hybridisation. Raw data with a global background subtraction were generated from GenePix pro 6.0. The analyses were done using the Acuity 4.0 software (Molecular Devices, CA, USA). The individual data sets were normalised at a log2 ratio value of 1. After normalisation, data were filtered to remove the spots flagged as not found. Only those spots with at least two replicates were considered. The gene expression of each strain was compared at both temperatures by performing biological triplicates. Significant genes with differential expressions were taken into account for further analysis using the SAM test [45] with an FDR below 5% using MeV software [46]. GO term analysis was performed with the online tools of SGD [47] or FunSpec [48] Database selecting for significant functional groups (p < 0.01) with Bonferroni correction for false positives. Data was deposited in Gene Expression Omnibus (GEO) Database with the accession number: GSE52545.

### Real-time qPCR

The PCR primers used in this study were TGGATTCCGGTGATGGTGTT - CGGCCAAATCGATTCTCAA for *ACT1* and TTCAATGCTGACAGAGACGCTATT – GATACGGACGGAAACAACTTCAC for *NSR1*. All the amplicons were shorter than 100 bp, which ensured maximal PCR efficiency and, therefore, the most precise quantification. RNA extraction was done as previously described (see above). A relative quantification model with kinetic PCR efficiency correction was built [49]. Experiments were carried out in triplicate. The control sample was extracted from the inoculum of *S. cerevisiae* and *S. kudriavzevii* at 28°C in the stationary phase after overnight growth in GPY. The reference gene used was *ACT1*, which showed excellent uniformity in the expression levels in these fermentation conditions [50], and all the reactions were done in a LightCycler® 480 Real-Time PCR System. Average of biological triplicates was calculated and standard deviations were lower than 20%. The gene expression levels are shown as the changes in the concentration of the studied gene as compared to the control sample and were normalised with the concentration of the housekeeping *ACT1* gene.

### Paromomycin assays

For the halo assays, yeast cells were grown overnight in GPY and diluted the next morning. They were then grown until the mid-log phase (approximately 1 × 10^7^ cells/ml) and then 175 μl were spread on each GPY plate. When the plate was absolutely dry, a filter (1 cm diameter) imbibed with different amounts of paromomycin (0.4, 2 or 10 μg of drug) was placed on the surface and plates were incubated at 30°C until the lawn was confluent. The measurement was taken from the point where colonies were grown. Inside the halo, there were only single cells and clumps. The assays were repeated twice.

### Translation rate determination

An initial concentration of 2 × 10^6^ cells ml^−1^, determined by absorbance at 600 nm, was used to inoculate 25 mL of synthetic must without methionine. Firstly 50 ml tubes were incubated at 12°C and 28°C, 10 μl of ^35^S-methionine (0.1 mCi, Hartmann Analytic GmbH, Germany) were added to the media and samples were taken at different time points. Next 2 ml of sample were mixed with 250 μl of 1 M NaOH in a glass test tube and were incubated at RT for 10 min. Two ml of cold TCA (25%) were added to the sample which was vortexed briefly. Samples were incubated on ice for 5 min. Vacuum filtration was used to collect the precipitated protein. 10% TCA pre-wet glass fibre filters were used to filter the sample with vacuum. Filters were rinsed 3X with cold 10% TCA and once with 95% ethanol to dry them and to prevent quenching. Dry filters were placed into scintillation vials with 2 ml scintillation fluid. Samples were measured in a scintillation counter after soaking the filters in scintillation fluid overnight. To determine the translation rate, cpm values were normalised with yeast growth (OD_600_). Translation rate was determined as the slope of linear regression calculation in normalised cpm versus the time graphs performed with the GraphPad Prism 5.03 software. Average and standard deviation was calculated from three independent biological replicates.

### Statistical analysis

Data were analysed with the Excel software. Results are expressed as mean and standard deviation. To evaluate statistical significance, two tailed *t*-student test was applied with *p*-value < 0.01. Bonferroni correction was used for transcriptomic and GO analysis.

### Availability of supporting data

The data set supporting the results of this article is available in the Gene Expression Omnibus (GEO) Database repository, GSE52545, http://www.ncbi.nlm.nih.gov/geo/query/acc.cgi?acc=GSE52545.

Also other data set supporting the results of this article are included within the article and Additional file 2: Table S2, Additional file 3: Table S4 and Additional file 4: Table S3.

## Electronic supplementary material

Additional file 1: Table S1: Yeast strains used in the present study and source where were isolated. (DOCX 14 KB)

Additional file 2: Table S2: Differential gene expression comparing *S. kudriavzevii* (12-28°C) with *S. cerevisiae* (12-28°C) response. (XLSX 27 KB)

Additional file 3: Table S4: Results from FunSpec database comparing *S. kudriavzevii* (12-28°C) with *S. cerevisiae* (12-28°C) response. (XLSX 15 KB)

Additional file 4: Table S3: Differential gene expression comparing 12 with 28°C in *S. kudriavzevii* and *S. cerevisiae.* (XLSX 305 KB)
