# Supplementary material for: Transcriptomics of cryophilic Saccharomyces kudriavzevii reveals the key role of gene translation efficiency in cold stress adaptations
Source: BMC Genomics. 2014 Jun 4;15(1):432. doi: 10.1186/1471-2164-15-432 (PMC4058008; doi:10.1186/1471-2164-15-432)
Supplement: Supplementary file 1 — Additional file 1: Table S1: Yeast strains used in the present study and source where were isolated. (DOCX 14 KB) [file 12864_2013_6110_MOESM1_ESM.docx]

**Supplementary Table 1.** Yeast strains used in the present study and source where were isolated.

| Species | Strain | Origin | Reference^†^ |
| --- | --- | --- | --- |
| *S. cerevisiae* | Lalvin T73^C^ | Wine fermentation (Spain) | T73 |
| *S. cerevisiae* | Lalvin QA23 ^C^ | Wine fermentation (Portugal) | QA23 |
| *S. kudriavzevii* | IFO 1802^T^ | Decayed leaf (Japan) | IFO1802 |
| *S. kudriavzevii* | CR85 | Oak Tree (Spain) | CR85 |

^†^ Yeast reference used in the present work, ^T^ Type strain, ^C^ Commercial strains.
